# Utilizing social media for community risk communication in megacities: analysing the impact of WeChat group information interaction and perception on communication satisfaction during the COVID-19 pandemic in Shanghai

**DOI:** 10.1186/s12889-024-19276-1

**Published:** 2024-07-15

**Authors:** Yasai Chen, Yiru Chen, Shan Yu, Shuni Yu

**Affiliations:** 1 Film-Television and Communication College, Shanghai Normal University, Shanghai, 200234 China; 2 The Center for Urban Culture Studies, Shanghai Normal University, Shanghai, 200234 China; 3 Department of medical oncology, Zhongshan Hospital, Fudan University, Shanghai, 200032 China

**Keywords:** Core residents, Community risk communication, Mega-city governance, Public health crisis, Risk communication, Social media

## Abstract

**Background:**

Against the backdrop of the global public health crisis, the COVID-19 pandemic has exposed significant disparities in the supply and demand of risk information related to public health crises, posing severe challenges to risk governance in megacities. Shanghai, China, introduced community WeChat groups for community communication, effectively facilitating the dissemination and response of grassroots information and providing a new path for interactive governance in the community.

**Methods:**

This study collected 1006 questionnaires from residents of 350 communities in Shanghai through an online survey between June 10 and July 10, 2022. Multiple linear regression analysis was conducted to examine the impact of different participants (including the community, core residents, and the combined community and core residents) on community risk communication, perceived communication quality, and dissemination themes related to COVID-19 on community communication satisfaction. Additionally, in-depth interviews were conducted with 20 core residents from different types of communities, focusing on the specific methods of risk communication through community WeChat groups and their ability to disseminate information, respond to, and solve problems.

**Results:**

Perceived information coverage and perceived response efficiency are significantly positively correlated with communication satisfaction. Notably, the speed of community information response has the greatest impact on communication satisfaction. Regarding COVID-19-related information dissemination themes, “community outbreaks, supplies, nucleic acids, outbreak prevention measures, and scientific content” all have a significant impact on communication effectiveness, with “nucleic acid testing information” having the greatest impact. Although the statistical data indicate that the participation of core residents in risk communication does not significantly affect communication satisfaction, it seems to be related to the size of the community, and the interview results further validate this conclusion.

**Conclusion:**

In the future, grassroots communities should consider the affordances of social media, recognize the significant correlation between risk communication and grassroots trust, and formulate more detailed and targeted risk communication strategies. In particular, incorporating core residents into “semiformal” grassroots organizations can improve community service quality, thereby enhancing community resilience in the face of public health emergencies.

## Background

With the development of urbanization and globalization, the world has evolved from a system of nation-states into a system of global cities [1]. In particular, the status of megacities as fundamental spatial units of the globe has become increasingly prominent. Cities such as Tokyo, Hong Kong, and London have achieved stronger social attractiveness and inclusiveness. However, these cities also exhibit characteristics such as rapid mobility, population diversity, and social stratification, making them reference points for both urban and global governance [2]. This evolution has led to significant challenges in community communication within megacities, especially since the outbreak of the COVID-19 pandemic [3]. Taking Shanghai, China, in 2022 as an example, the pandemic exposed significant disparities in the supply and demand of risk information related to public health crises. This has once again verified the following four major characteristics of social risks in megacities: spillover, amplification, superposition, and chain effects [4]. First, as an international metropolis, Shanghai’s pandemic situation, fuelled by media coverage, public attention, and population concentration, can lead to national and even global spillover and amplification of risks. Additionally, the implementation of public policies such as “home quarantine” has affected the operation of life and production in Shanghai, significantly increasing residents’ perception of secondary risks. This is directly reflected in the emotional nature of public opinion, which in turn leads to the dilemma of community risk response facing dysfunction or disorder. Therefore, in megacities where digital technology is highly prevalent, utilizing social media to communicate risks beyond spatial and temporal limitations can effectively enhance the resilience of community public service networks [5]. This includes the static stability of communities’ self-defense against risks, as well as the dynamic adaptability to respond flexibly as risks evolve [6].

In China, WeChat has become the daily communication choice for more than 1.3 billion people due to its free and convenient features and high response rate. With its strong connectivity and personal network-based dissemination characteristics, WeChat has become the primary medium for Chinese netizens to communicate risks with family and friends [7]. Especially driven by the urgency of the COVID-19 pandemic, community WeChat groups, which are public virtual communities formed by residents and grassroots workers using the WeChat platform, have been widely popularized in the grassroots governance of megacities. These groups have gradually evolved into the exclusive medium for grassroots community organizations to update COVID-19 risk information and communicate and respond to public needs, effectively enhancing government credibility and social stability. Furthermore, China’s grid-based management^1^ has been identified by several scholars as playing a critical role in the prevention and control of epidemics [8]. The governance framework includes the establishment of a “dual-line service” approach of “offline building of grids, online strengthening of the network”. Residents, through the WeChat platform, can “online call” the community full-time grid operator so that it can quickly coordinate the grid force to solve the problem. However, this community governance through online response has also given rise to a group of “core residents” who often play a prominent role in community communication, effectively mitigating the emotional imbalance of community residents due to “risk uncertainty” [9]. Residents of the community benefit from having core residents present, as they gain access to valuable information and emotional support that promotes social interaction. The need to train semiformal staff to effectively support the front line in addressing and resolving the information requirements and requests of the public has become apparent. This training is necessary to facilitate the engagement of various community stakeholders in community governance.

This study draws on research related to risk perception and interactive communication, focusing on community WeChat groups as the primary medium for grassroots communication during public health emergencies. Using multiple linear regression equations, this study analyses the factors influencing residents’ communication satisfaction and explores the relationships among community risk communication participants, communication themes, perceived quality of communication, and communication effectiveness. Based on the data, this study also discusses the opportunities and challenges that grassroots communities may face in using community WeChat groups for public health monitoring, risk communication, and management.

## Literature review and theoretical framework

### WeChat-based community communication

Academic understanding of risk communication has evolved from a top-down, one-way flow of information to emphasize two-way or multiway interactive communication between those who possess professional knowledge and those who lack relevant information [10], which refers specifically to the timely and continuous dissemination of risk-related information among government agencies, media outlets, subject matter experts and the general public. Therefore, during public health events such as COVID-19, which are highly infectious and rapidly spreading and have significant impacts, community communication is key to effective governance [11] and is a tool for developing targeted communication strategies and providing residents with accurate and timely information during public health emergencies [12]. Although community communication in China is characterized by the state’s “flexible control” of public affairs [13], the development of the internet society, where social media has become the dominant channel for Chinese citizens to access and exchange information [14], has contributed to a shift away from a closed and hierarchical system of community communication in China. Community managers are able to utilize community groups on WeChat to serve as “information publishers” and provide timely, credible, and transparent risk information to prevent “information harm” or “secondary harm” [15].

However, unlike Weibo, which is similar to Twitter in its weak connections and open social media structure [16], WeChat builds a multilevel dissemination flow based on strong connections within private circles. This emphasizes the use of intermediate relationships between familiar (personal relationships) and unfamiliar (public) individuals. This creates a chain effect in information diffusion, maximizing the effectiveness of communication [17]. For Chinese society, acquaintances or private circles are defined around the individual^2^, unlike the clear distinctions between public and private domains in Western culture. This cultural habit has been inherited in WeChat, distinguishing it from tools such as QQ groups (which focus on interest-based socialization and resource sharing) [18] and WhatsApp (which emphasizes data and privacy protection) [19]. WeChat’s dissemination reflects the perfect utilization of social capital, where the center of its communication relies not on the affordances of technology or platforms but rather on the individual’s power within the actor network [20]. During sudden events such as the COVID-19 pandemic, which are strongly associated with personal life and health, people use pandemic-related information as a social currency. They use the social relationships of “semifamiliar” contacts in WeChat friends circles as a bridge, frequently communicating and exchanging information to update their own risk perception, which serves as a basis for their risk-related decisions. Additionally, due to the massive population mobility in China’s megacities^3^, the traditional understanding of “distant water cannot quench present thirst; a distant relative is not as good as a near neighbor” persists among Chinese people who have left their hometowns. Grassroots organizations, as representatives of grassroots public authority, naturally foster a sense of collective identity among social members. Supported by this geographical and public nature, the emergence of “community WeChat groups” combines the advantages of systemic trust and interpersonal trust [21]. Therefore, basic social cultural norms that emphasize community support and cohesion can support the establishment of resilience. This approach can encourage the development of social capital while also mitigating the potential losses caused by conflicts during periods of community deprivation [22]. This provides a new approach to the management of public health emergencies. By creating virtual communities, WeChat has directly facilitated both vertical information distribution and horizontal interaction among neighbors. Grid management [8] has greatly changed the dynamics of community governance, favouring information iteration and timely communication. More importantly, it has fostered a positive atmosphere of public trust [23, 24], thereby reducing the likelihood of secondary disasters resulting from risk amplification [25, 26].

Based on the above discussion, there is a consensus that utilizing community or social media for risk communication can promote the management of public health events. This study focuses on the emerging and integrated communication medium of “community + social media” and delves into how WeChat communities enhance communication effectiveness from the perspective of digital communication as social and psychological support.

### Core residents in information dissemination

The term “core” is a concept originating from China that encompasses the notions of “core farmers,” “core cadres,” and “core youth” [27]. The term “core residents” is introduced within the framework of community governance. The aforementioned entity plays a crucial role in the participatory aspect of community grid governance. The term “community residents” refers to individuals who derive their primary income or sense of accomplishment from serving the community. They actively participate in community administrative affairs, oversee public matters, and offer support within the neighborhood [9]. Core residents can be classified into three distinct categories. The first category comprises grassroots workers who serve as “block leaders”^4^. These individuals engage in direct communication with community residents through the community WeChat group, addressing residents’ concerns, disseminating information, and resolving any issues that may arise. The second category consists of volunteers who participate in community management as “building leaders^5^“. They play a crucial role in disseminating information about the epidemic within the community and facilitating communication among other building leaders regarding the specific needs of residents in each building. The third category comprises dispersed individuals who assist grassroots organizations in reducing their workload through their involvement in tasks related to building collection, the dissemination of community information, and the coordination of group purchases for daily essentials. The community serves as the fundamental entity of social governance, and there is a shift in grassroots governance from production governance to life governance [28].

It has been pointed out that the space created by social networks is itself akin to a “community”, with many influential nodes coming from ordinary citizens who can be utilized by the public sector as a mobilizing force for discursive activism [29]. The “core residents” of online community groups are important actors in community mobilization after the virtualization of grassroots communication platforms. Residents’ engagement in the community exhibits three key characteristics: the public nature of their goals, the subjective nature of their role, and the sustained nature of their participation. In the context of the epidemic in Shanghai, the overwhelming burden on communities resulted in the organic involvement of numerous community residents who united in their efforts to combat the outbreak. This collective action gave rise to a novel “perception and representation” of risk, which was expressed through online mutual assistance and was a manifestation of the strengthening of community resilience through social capital [30]. Core residents, who exhibit a preference for utilizing social media platforms as a means of self-expression, demonstrate a dualistic approach encompassing both professional and scientific perspectives, as well as emotional considerations, in their representation of information. This multifaceted approach effectively caters to the public’s demand for both informative content and emotional support. They frequently assume the role of steadfast advocates for public matters, collectors of community sentiment, and suppliers of everyday amenities. They exhibit a strong commitment to supporting the community and play a central role in fostering community resilience.

In conclusion, core residents play an integral role in the community by leveraging their social connections to engage with vulnerable networks and bridge the gap between community organizations and these marginalized populations [31]. Shah et al. demonstrated that during the COVID-19 pandemic, interpersonal risk communication had a more significant impact on individual trust and preventive behavior than did media risk communication [32]. Moreover, residents’ understanding of risk information is influenced by their trust in the information source [33]. These studies suggest that core residents, utilizing their inherent trust advantages, can participate in risk communication not only to enhance the effectiveness of communication but also to provide new pathways for community risk management. During the Shanghai epidemic, three types of communication subjects existed—the community or core residents were directly involved or a multifaceted communication subject in which the community and core residents were jointly involved—and these three types of communication subjects created three types of communication in the WeChat community that may have had an impact on resident satisfaction (H1). Further subhypotheses are proposed in this regard:

H1-1: The communication model that involves core residents directly in risk communication results in greater resident communication satisfaction than does the direct community communication model.

H1-2: Core resident involvement in community communication can significantly influence community communication satisfaction.

Additionally, the study will combine questionnaire surveys and in-depth interviews to create a demographic profile of core residents, further explaining the role they play in community risk communication.

### Community information response and risk perception

The information flow in risk communication is a closed loop, and one-way community information transmission alone cannot resolve all community risk conflicts. Xu et al. introduced the concept of information feedback into this cycle, emphasizing dialogue and communication between the government and the public to promote orderly community governance [34]. In recent years, Beijing, Shanghai, and other megacities in China have started to explore feedback mechanism reforms. Li introduced a feedback mechanism that utilizes the response rate, satisfaction rate, and problem-solving rate as key indicators to enhance governance in megacities [28]. This indicates that a positive feedback mechanism also needs to consider the resolution of individual issues, proactive governance, and effective management of complaints. In the case of public health emergencies such as an epidemic, residents are more proactive in obtaining information, their information needs are more concentrated and intense, and they have more urgent problems to solve [35]. They have a stronger sense of questioning and criticizing the actions taken at the grassroots level. Therefore, it is particularly important for grassroots communities to actively respond to public information needs during pandemics and maintain grassroots credibility. Failure to meet these demands may result in widespread cyber-opinion and potential conflict, and fostering these negative sentiments may make it difficult for grassroots communities to implement risk management [36].

**Fig. 1:**
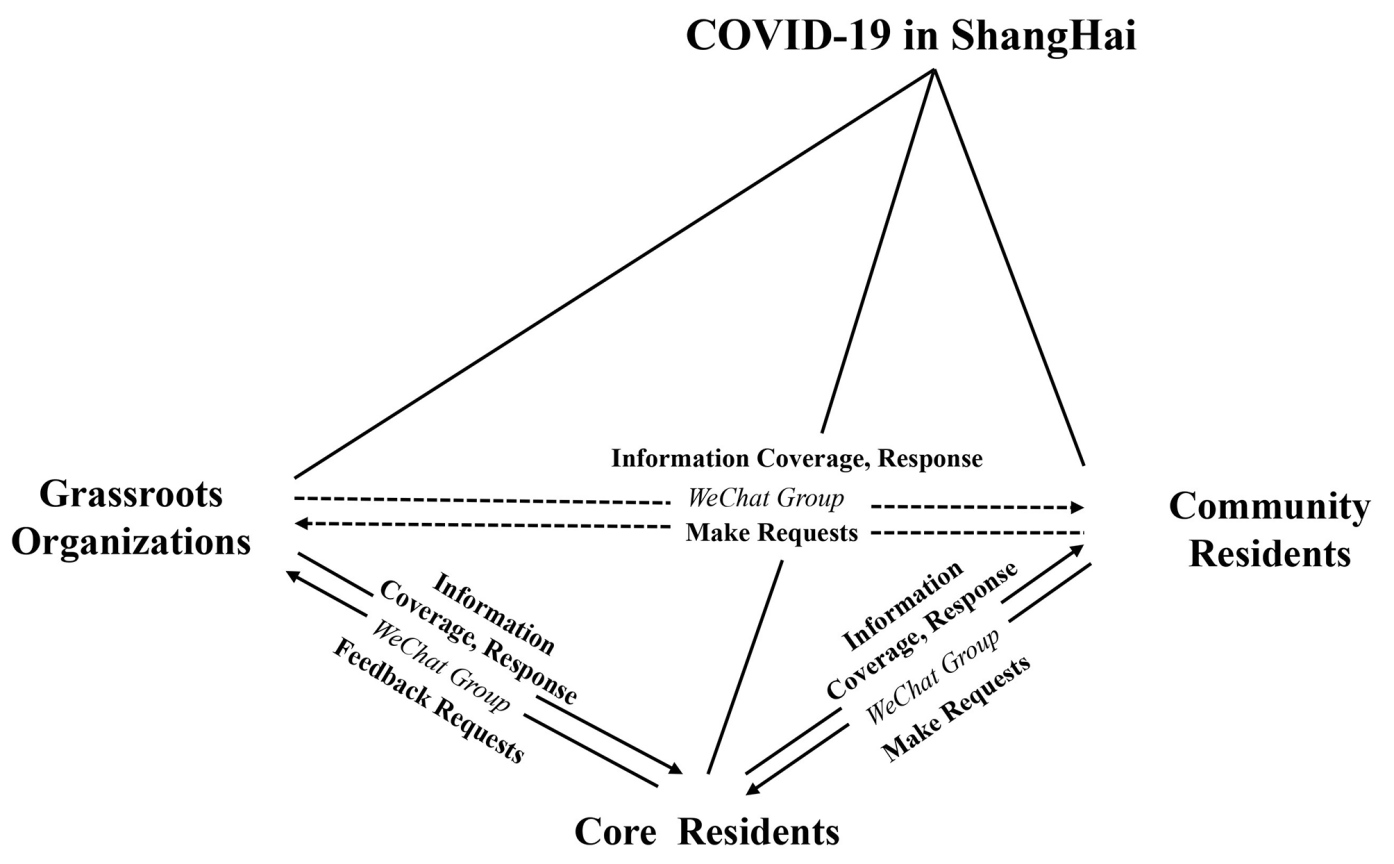
Kite-type coorientation of risk communication in communities in Shanghai

This study argues that the information dissemination behavior of community WeChat groups is essentially an “interactive dialogue,” and its distinctive feature is an instantaneous response in the form of questions, complaints and answers. Moreover, proactive and rapid service responses have the ability to maintain trust and soothe emotions. These advantages of digital communication have made community WeChat groups a widely used interactive communication and governance medium for grassroots organizations in megacities during the COVID-19 pandemic, greatly enhancing community response and governance efficiency [37]. According to Kooiman and Bavinck, interactive governance is a multifaceted phenomenon characterized by the interaction of various social and political actors who possess divergent interests [38]. This process aims to facilitate the resolution of social issues and the generation of social opportunities through effective communication, mobilization, and the establishment of interactive norms. The concept discussed here is the process of negotiating and reconciling the conflicting interests of various stakeholders through effective communication and negotiation. This approach differs from the traditional top-down model of “acceptance of authority” and instead emphasizes the involvement of multiple actors in collaborative and coordinated actions [39]. To address the possibility of conflicting cognitive views between third parties or two individuals, McLeod et al. proposed the “Kite model,” which states that social elites, the media, and the public have a mutual relationship concerning social issues and related perceptions and attitudes [40]. Therefore, this study introduces a kite model of risk communication that encompasses the communities, core residents, and residents of Shanghai (Fig. 1).

Social media is increasingly integrated into the risk communication strategies of public health and emergency agencies [41]. Due to complex demographic characteristics and immense population mobility and size in megacities, controlling this situation has become challenging. To respond to sudden new outbreaks or to implement special lockdown policies, almost all communities have established community WeChat groups. Initially, these groups were promoted to ensure that government agencies or grassroots organization personnel could achieve real-time dissemination and timely responses. Later, community residents adopted them as a community for emotional dialogue and information exchange. Core residents, in particular, have taken on a public role, acting as intermediaries to facilitate the upwards and downwards flow of information and meet diverse communication needs. This communication method is widely implemented only in Chinese megacities, breaking through the limitations of previous unidirectional and time-space restricted linear information flow and establishing a closed-loop information communication model that incorporates a community of multiple actors. Additionally, the effectiveness of this interactive communication model is reflected not only in the improvement of communication efficiency but also in the facilitation of the implementation of public health emergency management mechanisms. Real-time responses shape residents’ perception of “effective communication,” becoming an important foundation for group decision-making and risk assessment.

Previous research has shown that the rapid dissemination of social media across time and space can amplify or even create environments of risk perception bias among individuals or within groups [42]. Once there is a severe mismatch between subjective environmental risk perception and the likelihood of objective environmental risk exposure, the resulting sense of crisis can trigger a chain reaction at the societal level, hindering risk response efforts [43]. Therefore, the exploration of “risk as feeling,” including the roles of emotion and affect, is regarded as a critical cue in determining risk judgment and decision-making [44] and is becoming a central focus in risk communication research [45]. The level of satisfaction with community communication [46] can not only test whether the content and methods of risk communication are effective for residents but also reflect the overall emotional perspective of residents towards community response and management efforts. It is one of the strong indicators for measuring the effectiveness of risk communication [47, 48].

Therefore, this study focuses on the specific dissemination content and communication methods of WeChat communities in Shanghai during the COVID-19 pandemic. From the perspective of audience psychological perception, this study discusses residents’ perceived quality of community risk communication (including perceived information coverage and perceived response efficiency) and the correlation between specific themes of information dissemination and communication effectiveness. Specifically, the frequency and adequacy of pandemic-related information dissemination are the foundation for measuring community communication satisfaction, and the theme on which risk information is disseminated may affect residents’ risk perception and thus have an impact on emotions and actions [41, 43], especially during a blockade, when material information may be critical to maintaining daily life. Additionally, the speed of information response, the frequency of information responses, and the frequency of receiving feedback after problem resolution play crucial roles in evaluating the level of grassroots proactive feedback, which are key parameters that influence satisfaction.

H2: The perceived quality of community communication impacts resident satisfaction in the WeChat community. The subhypothes is proposed in this regard is:

H2-1: The frequency of receiving feedback after problem resolution is the most significant factor influencing communication satisfaction.

H3: The dissemination of COVID-19-related information impacts resident satisfaction in the WeChat community. The subhypothes is proposed in this regard is:

H3-1: Information on material supplies is the most important theme influencing communication satisfaction.

## Data and methods

### Procedure and participants

The data were collected from an online questionnaire survey conducted from June to July 2022 in Shanghai, China. The study population comprised residents living in communities in 16 administrative districts in Shanghai who had joined the community WeChat group. After referring to previous international studies on sample sizes in large Chinese cities [49, 50] and taking into account the 10% invalid questionnaire recovery rate, we set the sample size to be approximately 1,000. In the data collection phase, we mainly used random sampling and posted online questionnaires in Shanghai community WeChat groups via the “Wenjuanxing” platform (a commonly used online survey tool in China). We ultimately received a total of 1006 questionnaires, and the survey results covered 100% of Shanghai’s administrative districts, 97% of its streets, and 25% of its residential community committees. After excluding invalid questionnaires, we analysed 921 valid questionnaires. The demographics of the survey respondents are shown in Table 1.

Furthermore, to gain a more comprehensive understanding of the specific practices of risk communication within WeChat communities at the grassroots level, this study conducted semistructured online in-depth interviews with a total of 20 core residents in Shanghai, highlighting the advantages of utilizing social media for public health information dissemination and the realities of the dilemmas of grassroots management to obtain additional findings. In addition to collecting basic information from the respondents, such as age, gender, education level, and area of residence, the questionnaire was structured into five sections. These sections aimed to gather data on various aspects related to the establishment of the community WeChat group. The primary inquiries, their corresponding content, and the statistical values are presented in Table 2.

**Table 1:** Summary statistics of the sample structure [*N* = 921]

Variables	Classification	Number of People	Percentage	Variables	Type Classification	Number of People	Percentage
Gender	Male	403	44.48%	Educational Background	Masters and above	307	33.34%
	Female	518	55.52%		Undergraduate	377	40.93%
Age Distribution	Under 18 years old	8	0.80%		Junior college	102	11.07%
	18–25 years	129	14.00%		High School and below	135	14.66%
	26–30 years	118	12.80%	Size of Neighborhood	Under 300 people	86	9.34%
	31–40 years	320	34.70%		300–500 people	71	7.71%
	41–50 years	211	22.90%		501–1000 people	80	8.69%
	51–60 years	75	8.10%		1000–3000 people	312	33.88%
	60 + years	60	6.90%		3001–5000 people	147	15.96%
					Over 5000 people	225	24.43%

**Table 2:** Main questions in the questionnaire and the design of each variable

Main Content	Title	Statistical Values
Establishment of the community WeChat group	Was the community WeChat group (owners’ groups, residents’ groups, building groups) established before or after the outbreak?	Before the outbreak, after the outbreak
	During the epidemic control period, did the staff of the street committee join the “community WeChat groups”	Yes, No, Not sure (“No” and “Not sure” mean the questionnaire access will be terminated)
	Whether the community WeChat group was formed by staff of the street residence committee?	Yes, No, Not sure
Ways to participate in the community WeChat group	How do the residents’ committees participate in the dissemination of information in the community WeChat group?	“1” denoted “direct participation,” “2” represented “indirect participation through building managers,” and “3” indicated “both.”
Perceived Information Coverage	How often do the residents’ committees disseminate information directly or indirectly through the community WeChat group?	Five statistical values from “not published” to “frequently published”
	What is the adequacy of the information disseminated by the residents’ committees directly or indirectly through the community WeChat group? What are some of the topics for posting information? Divided into five categories, namely, information on community outbreaks, information on supplies, information on nucleic acids, outbreak prevention measures, and scientific content.	Options are multiple choice, with more options representing greater informational adequacy, cumulative scoring with a maximum score of 5 points.
Perceived Response Efficiency	How quickly do the residents’ committees respond to questions and queries raised directly or indirectly through the community WeChat group?	Five statistical values from “very slow” to “very fast”
	How often do the residents’ committees respond to questions and queries raised by the residents either directly or indirectly through the community WeChat group?	Five statistical values from “never respond” to “always respond”
	How often do the residents’ committees give feedback through the community WeChat group after solving problems?	Five statistical values from “no feedback” to “frequent feedback”
Communication Satisfaction	How satisfied are you with the information communicated by the residents’ committees through the community WeChat group?	Five statistical values from “very dissatisfied” to “very satisfied”

#### Dependent variable: community communication satisfaction

Subjective satisfaction with communication is primarily used as a variable to measure the effectiveness of communication skills and methods in health communication studies [51, 52]. Although there are similar studies involving community communication [46], they do not specifically address the content and methods of communication. To address these gaps, this study uses the satisfaction indicators of residents interacting via WeChat communities during the Shanghai pandemic as the dependent variable. It further discusses the impact of different communication participants (including the community, core residents, and the combined participation of the community and core residents), communication content, and the quality of dissemination and response on the effectiveness of community communication. Residents responded to the question “How satisfied are you with the information communicated by the residents’ committees through the community WeChat group?”

#### Independent variables

In the present study, three distinct independent variables were identified: participation in governance by core residents, the perceived quality of community risk communication and COVID-19-related risk communication.

To examine the involvement of core residents in governance, this study posed the following question: “In what ways do grassroots organizations engage in the dissemination of information in the community WeChat group?”

To verify which perceptions of risk information communication affect communication satisfaction, this study designed a community risk communication quality perception scale based on specific communication scenarios. This scale references the design of service quality perception variables by Xie et al. [46] and the feedback mechanism indicators proposed by Li et al. [28]. It is divided into two aspects: perceived information coverage and perceived response efficiency. Perceived information coverage includes two factors: the frequency and adequacy of pandemic-related information dissemination. Perceived response efficiency includes three factors: the speed of the information response, the frequency of the information response, and the frequency of receiving feedback after problem resolution. The response options ranged from “1”, indicating a very slow response, to “5”, indicating a very fast response. The Kaiser–Meyer–Olkin (KMO) value was 0.78, indicating that the data used in the analysis were suitable for factor analysis. The study conducted exploratory factor analysis (EFA). The rotated five-factor component matrix was extracted into two main components, consistent with the study design. The cumulative variance after rotation was 86.18%, and the factor loadings ranged from 0.842 to 0.925. Furthermore, the study conducted a confirmatory factor analysis (CFA). Model fit was evaluated based on the most commonly used criteria in the literature: a confirmatory fit index (CFI) and normed fit index (NFI) higher than 0.90 and a root mean square error of approximation (RMSEA) lower than 0.08 indicate a reasonable model fit. CFA showed that this model was acceptable (χ²/df = 2.14, CFI = 0.98, NFI = 0.97 RESEA = 0.07), and the Cronbach’s alpha reliability coefficient of the scale used in this study was 0.92, indicating that the scale had high internal consistency.

Finally, investigating the impact of different COVID-19-related information themes on communication satisfaction is conducive to communities formulating better information dissemination plans in the future. This study categorizes the main pandemic information dissemination themes into five categories: information on community outbreaks, information on supplies, information on nucleic acids, outbreak prevention measures, and scientific content.

#### Control variables

Individuals exhibit variations in their subjective assessment of risk, with women generally perceiving higher levels of risk than men [53]. Older individuals who are well educated tend to have a lower perception of risk [33]. Therefore, we implemented measures to account for the sociodemographic attributes of the participants, such as gender, age, and level of education.

## Data statistics and analysis

### Descriptive statistics

In this study, the average satisfaction with the risk information score was 2.70, indicating that all community respondents in our sample rated community risk communication on average.

In relation to the dissemination of community information, a significant majority of respondents (61.8%) expressed the belief that residents’ committees were more active in releasing relevant information through the community WeChat group. Additionally, a considerable proportion (57.1%) of respondents indicated that they found the information released to be sufficient. The content primarily consisted of information on nucleic acids (84.8%), outbreak prevention measures (45.8%), community outbreaks (49.1%), medications, anti-fraud measures, group purchase suppliers, and responses to inquiries regarding the purchase of medicine for specific groups. The content effectively addressed the fundamental information requirements of community residents amidst the epidemic by consolidating information from diverse departments, including medical care and disease control. Notably, 40.6% of the residents felt that the frequency of relevant information shared was low, while 42.9% believed that the information provided was insufficient. These residents noted that the grassroots community proactively released information solely on nucleic acid, while other types of information, such as updates on drug purchases, were shared passively.

**Table 3:** Comparison of the satisfaction of different subjects participating in communication

Classification	Community		Core Residents		Community + Core Residents	
	M	SE	M	SE	M	SE
All communities	3.32	0.91	2.97	1.19	2.25	1.11
Small-scale communities	3.75	0.94	2.00	1.16	3.09	1.21
Medium-scale communities	2.95	1.13	2.25	0.89	3.16	1.36
large-scale communities	3.16	1.20	2.58	1.03	3.24	1.27

Note: M = Mean; SE = Standard Error of the Mean. Scores ranged from 1–5; higher scores indicate higher levels

#### Comparison of the communication satisfaction of core residents participating in Community Risk Communication

Table 3 shows a comparison of satisfaction with participation in communication by different subjects (direct community communication, direct core resident communication, and community and core resident multiple communication models). The study findings demonstrated that residents expressed the highest level of satisfaction with the direct information communication approach used by community organisations, with an average score of M = 3.32. Credible information exchange is proven to effectively break hierarchies and ensure timely and effective dissemination of information related to an epidemic.

Among the three communication models developed, the second model involves only core residents, and the third model includes both community organisations and core residents, which are probably mainly used in medium and large communities. During public health emergencies, the emergence of social media such as the WeChat community has increased the convenience of communication but has subsequently caused problems such as unfocused discussions, redundancy of information and fragmentation of information. The implementation of new communication methods with the active participation of core community members has the potential to reduce the burden of community workers to some extent and enhance the efficiency of information dissemination. However, residents’ satisfaction with risk communication by core residents (M = 2.97) was not as high as their satisfaction with direct community communication (M = 3.32); thus, H1-1 was not successfully verified. This suggests that the effectiveness of communication may be influenced by the size of the community. Therefore, the study further selected and compared satisfaction data from residents of communities of different sizes to substantiate this possibility. In large and medium-sized communities, the communication satisfaction scores are ranked as follows: the mean satisfaction score for the diverse model involving both community and core resident participation is greater than that for community communication alone, which in turn is greater than that for the core resident communication model. Additionally, the larger the community is, the greater the likely satisfaction score for the diverse communication model.

However, to further understand how core residents participate in community communication, a study based on the risk communication kite model established in the literature review focused on the two aspects of residents’ information needs and community information response. The interviews were conducted with 20 core residents. The demographic data are shown in Table 4.

**Table 4:** Interview core resident demographics

No.	District	Age	Occupation	Education	Role	Local Resident
1	Hongkou	30	Enterprise Employee	Master’s Degree	Volunteer	Yes
2	Hongkou	45	Freelancer (Former Chemical Engineer)	Master’s Degree	Volunteer	No
3	Hongkou	57	Nurse	Vocational School	Volunteer	No
4	Hongkou	39	State-owned Enterprise Employee	Master’s Degree	Volunteer	No
5	Xuhui	42	Senior Engineer	Doctorate	Building Manager	No
6	Xuhui	50	State-owned Enterprise Employee	Bachelor’s Degree	Community Block Leader	Yes
7	Xuhui	36	University Teacher	Doctorate	Volunteer	No
8	Xuhui	24	Student	Bachelor’s Degree	Volunteer	Yes
9	Xuhui	60	Retired Employee	Vocational School	Volunteer	Yes
10	Putuo	52	Doctor	Doctorate	Volunteer	Yes
11	Putuo	38	State-owned Enterprise Employee	Master’s Degree	Community Block Leader	No
12	Yangpu	34	State-owned Enterprise Employee	Master’s Degree	Volunteer	No
13	Yangpu	41	University Teacher	Doctorate	Volunteer	No
14	Changning	45	Police Officer	Bachelor’s Degree	Volunteer	Yes
15	Changning	48	Enterprise Employee	Bachelor’s Degree	Volunteer	No
16	Huangpu	56	Private Enterprise Employee	Associate Degree	Volunteer	Yes
17	Huangpu	49	Self-employed	High School	Building Manager	Yes
18	Pudong	34	Foreign Enterprise Employee	Master’s Degree	Volunteer	No
19	Fengxian	43	Enterprise Employee	Bachelor’s Degree	Volunteer	Yes
20	Jinshan	46	Enterprise Employee	Bachelor’s Degree	Volunteer	Yes

* Local Resident: Shanghai native to the parents’ generation

Similarly, the interview results indicate that the effectiveness of core residents during the Shanghai pandemic may be related to the size of the community. In medium-sized and large communities, the participation of core residents in the communication process improves the effectiveness of risk communication and solves the dilemma of labour scarcity at the community level, but it also brings about information “distortion” and reduced feedback speed under the filter of indirect dissemination of information, which affects the satisfaction of residents with risk communication. In addition, due to the limited knowledge and attention span of community residents, there is a strong correlation between the credibility of the content of the message and the credibility of the source [54]. Therefore, grassroots organizations that demonstrate professionalism, knowledge and impartiality are more likely to earn the trust of the public compared to core residents, thus increasing the effectiveness of risk communication. However, in small communities, the direct dissemination of risk information by grassroots community managers through community WeChat groups has met the information needs of most residents, minimizing noise in the communication process. Therefore, residents’ perceptions of the effectiveness of core resident communication are not significant. This does not mean that the participation of core residents in small communities is ineffective.

The study’s interview data show that the core residents who participate in community communication are primarily young and middle-aged adults aged 20–55 years, most of whom have a high level of education^6^. Their main occupations include corporate employees from various industries, government officials, and students. The professional diversity of core residents allows them to effectively use their skills to assist community members. Among the 20 core residents interviewed, 2 were community workers (block leaders), 2 were building managers, and the remaining 16 were volunteers (dispersed individuals). The tasks of these three different types of core residents also vary significantly. Core residents who are community workers, due to the professional nature of their work, can contact key community managers more quickly, thereby taking on the primary roles of information dissemination and response within the community group. Building managers primarily facilitate the upwards and downwards flow of information. Because community staff are often short-handed and unable to communicate directly with residents, the tasks of consolidating information, publishing it, and collecting and responding to residents’ questions are handled by building managers. The proportion of the first two types of core residents is very low; most of them belong to the third type—volunteers—they generally assist building managers or community managers, often taking on assigned tasks, each performing their duties or fully utilizing their initiative and expertise to engage in closer communication and dialogue with community residents. For example, advertising professionals create flowcharts for group purchases of supplies, and engineer couples design plans for rapid one-hour nucleic acid testing for the entire building. Additionally, some core residents (such as interviewed merchants or local retired employees in Shanghai) may have strong social capital in Shanghai, enabling them to quickly gather “inside information” on material procurement and supply. This rapid exchange of information compensates for the potential lack of specificity or delays in information dissemination by the government or traditional media.

Additionally, as a megacity, Shanghai’s complex population composition leads to a tendency towards community diversity. For example, international communities primarily composed of foreign friends exhibit significant cross-cultural differences, while communities with severe aging issues find it difficult to implement control measures directly through the internet. Each community needs to adopt personalized governance methods, and core residents significantly compensate for the lack of labor. They cooperate with the residents’ committees, playing a strong coordinating role in information interpretation and risk communication. Moreover, the involvement of diverse actors in community interactive governance benefits residents not only from the perspective of information dissemination but also, more importantly, through the formation of a community triggered by a crisis, awakened by national consciousness, and connected through emotional interactions. Under this triple identity resonance, residents can balance their risk perceptions through interpersonal risk communication, reinforcing their trust in grassroots organizations.

#### The impact of the perceived quality of risk communication (perceived information coverage and perceived response efficiency) on community communication satisfaction

To test Hypothesis 2, we constructed several models using multiple linear regression analysis (Table 5). Model 1 includes the satisfaction model constructed from perceived information coverage and perceived response efficiency. Model 2 adds control variables (age, gender, and education level) to Model 1. Additionally, to reflect the influence of core residents’ participation in community communication on communication satisfaction, the study also includes the variable of core residents’ participation in Model 2.

The model data show that demographic variables and the participation of core residents did not have a significant impact on the statistical results, and H1-2 is not supported. The results of regression analyses (Models 1 and 2) all show that perceived information coverage and perceived response efficiency have a positive predictive effect on residents’ satisfaction. The overall impact of perceived response efficiency is more significant than that of perceived information coverage. This indicates that in responding to public health emergencies, the core factor determining whether the risk communication of grassroots organizations meets the public’s information needs is rapid response. In a semiclosed community WeChat group, the time lag in information response can amplify residents’ anxiety about the instantaneous “wait” created by social media; therefore, if residents’ issues are not addressed in a timely manner, the effectiveness of information communication will be affected. If residents’ dissatisfaction is expressed in the group, it will form emotional resonance with other members, which will ultimately weaken the power of the grassroots organisations’ discourse and communication efficiency [55]. At the same time, the data indirectly reflect that the convenience of social media-based communication methods has increased residents’ expectations for communication quality. They are no longer satisfied with mere information dissemination but emphasize the flexibility and humanization of management strategies [56]. In particular, the speed of community information response (B = 0.35) plays a crucial role in the effectiveness of risk communication, and insufficient information and untimely feedback have the potential to trigger new online rumours [57]. Therefore, “debunking rumours” have become an especially important aspect of risk communication during emergencies [58]. Moreover, both the frequency of feedback after problem resolution and the speed of information response have a greater impact on public satisfaction. This shows that residents’ response demands also push grassroots communities to respond positively, forming a virtuous cycle. However, the actual survey results indicate that grassroots community information responses still need to be strengthened. Some grassroots cadres have a weak sense of public service, limited ability to optimize interactive communication through network technology, and insufficient emergency response and organizational capacity [59]. Unproactive response and shirking of responsibilities by streets and neighborhood committees are more common in risk communication. Moreover, although the incorporation of social media into risk communication ensures information dissemination and response to the greatest extent possible, in the face of public health emergencies, there may be a realistic contradiction between the public health department’s inability to provide immediate action guidance and the residents’ urgent need for information, which creates a very high standard for grassroots community leadership in carrying out targeted actions in response to changes in the situation.

**Table 5:** Results of regression analysis on community communication satisfaction in Shanghai grassroots communities

Independent Variable		Model 1			Model 2		
		B	SD	*p*	B	SD	*p*
Perceived Information Coverage	Frequency of information release	0.13	0.03	< 0.001	0.13	0.03	< 0.001
	Adequacy of information dissemination	0.10	0.02	0.001	0.11	0.02	< 0.001
Perceived Response Efficiency	Speed of information response	0.35	0.04	< 0.001	0.36	0.05	< 0.001
	Frequency of information response	0.14	0.05	0.002	0.15	0.05	0.001
	Frequency of feedback after problem solving	0.31	0.04	< 0.001	0.30	0.04	< 0.001
Communication Subjects	Participation of Core Residents				-0.02	0.05	0.358
Control Variables	Age				0.00	0.02	0.98
	Gender				0.02	0.05	0.339
	Academic qualification				0.04	0.02	0.101
	R²	0.83			0.84		

Note: B = standardized coefficient; SD = standard deviation

Additionally, perceived information coverage, the frequency of information release (B = 0.13) and adequacy (B = 0.10) had an impact on the level of satisfaction that residents experienced with communication. This finding is consistent with previous findings that timely, proactive, and adequate disclosure of epidemic information during public health emergencies is crucial for the collaborative efforts of community organizations, community residents, and social mobilization [12]. Among them, the adequacy of information (information on community outbreaks, information on supplies, information on nucleic acids, outbreak prevention measures, and scientific content) is positively correlated with communication satisfaction, partially confirming Hypothesis 3. To further discuss the impact of different pandemic information themes on residents’ perceived communication effectiveness, this study included five themes in the same regression model (Table 6).

**Table 6:** COVID-19 information dissemination themes and regression analysis

	M	SD	95% CI	B	t	*p*
Release of community outbreaks	0.49	0.12	(0.05, 0.52)	0.28(0.12)	2.34	0.020
Release of community supplies	0.60	0.13	(0.14, 0.63)	0.39(0.16)	3.08	0.002
Release of nucleic acids information	0.85	0.14	(0.57, 1.14)	0.86(0.26)	5.97	< 0.001
Release of the contents of outbreak prevention measures	0.46	0.14	(0.09, 0.62)	0.36(0.15)	2.64	0.009
Release of scientific content	0.24	0.14	(0.38, 0.93)	0.66(0.23)	4.65	< 0.001
R²	0.41					

Note: M = mean; SD = standard deviation; B = standardized coefficient; CI = confidence intervals

#### The impact of the dissemination of different pandemic information themes on community communication satisfaction

Information content plays a significant role in shaping individuals’ risk perceptions and “secondary public opinion,” often resulting from the alignment of organizational, media, and public agendas [60]. The greater the relevance of information dissemination topics to public concerns, the greater the public’s level of attention to information content related to relief and recovery, facts, and response guidelines. In this study, “community supplies” and “community outbreaks” were factual information topics, whereas “epidemic prevention measures” and “scientific information” were response guidelines. The results show that both types of information have a significant impact on communication effectiveness, which is inconsistent with previous research findings that guideline-type information does not significantly impact dissemination effectiveness [61]. This is because the COVID-19 pandemic is undoubtedly the most globally impactful public health emergency since the 20th century, overturning previous understandings of public health emergency control. First, community supply information was not the most influential theme (H3-1 was not supported), possibly because the survey phase was already in the later stages of the epidemic and supplies were no longer an important issue. However, nucleic acids information (B = 0.26), as a specific type of factual information, is directly related to residents’ lives and health as a form of public policy, making it the most concerning pandemic theme for grassroots communities and residents. Additionally, given the prolonged nature of the pandemic with no clear end date, people are particularly concerned about providing guidance on how to navigate the tense period of the pandemic and how to cope with COVID-19 in the future. Conversely, the frequent release of a single type of factual information by grassroots communities may lead to residents’ selective disregard or information fatigue. Furthermore, the data show that residents perceived a lack of dissemination of guidance information, especially popular scientific content (M = 0.24).

## Discussion

This study draws on risk perception and interactive governance-related theories to examine the impact of social media on community communication satisfaction during public health emergencies. Our contribution is to explore how mega-city grassroots communities in China conduct risk communication through community WeChat groups, particularly by leveraging the power of core residents. This study also investigated residents’ subjective satisfaction based on their perceptions of information coverage and response efficiency, thereby exploring effective paths for risk communication from the perspective of community service quality.

### Coresidents’ roles and tasks in WeChat community communication

This study reveals that the emergence of social media has broken the previous binary dialogue model of “top-down”, and a new communication form dominated by core residents has emerged in grassroots communities, which provides a new approach to grassroots interactive governance, socioemotional mobilization, and so on [62]. Although the results of the regression data show that the participation of core residents in risk communication does not have a significant effect on improving resident satisfaction and communication effectiveness, both the positive comments on the work of the “building manager” in public opinion and the approval attitudes held by the residents through our in-depth interviews reveal that the “core residents” have a significant impact on the community risk communication system.

In contrast, the inconsistency between the regression results and the research hypotheses—where core resident participation in community communication did not have a significant effect on resident satisfaction—suggests that in the actual community communication process, multilevel communication involving core residents lacks reliability. Residents need to verify the credibility of the information content and channels, which presents a hidden problem. This also indicates that grassroots organizations should incorporate the specific tasks undertaken by core residents into community management planning. For instance, as previously discussed, considering the issue of noise in indirect communication, core residents in small communities find it difficult to play a major role in information dissemination. In fact, the roles that core residents need to assume should be more clearly defined according to the different sizes and types of communities.

Professional Health Promotion. In the communication of community WeChat groups, it is advisable for communities to involve staff from relevant public health agencies as facilitators to coordinate multiparty communication efforts through frequent interactions within the community [63]. On the one hand, it may improve residents’ risk perception, and under the guidance of expert-led professional discourse, it may convert plain information dissemination into a dialogue-based exchange focused on residents and information [64]. On the other hand, when faced with the spread of rumours or public opinions, with professional endorsement, it becomes possible to address and resolve such issues at their root cause within community WeChat groups.

Nonprofessional community mobilizers. Community WeChat groups provide a platform for interaction and communication among grassroots organizations, core residents, and residents, which not only offers the possibility of a “one core, multidimensional” megacity community governance system but also helps residents to know the information and propose solutions in a crowdsourced manner [65, 66]. Therefore, in the future, mega-city grassroots communities can explore the possibility of involving core residents in the role of community mobile communicators and providing targeted information and feedback through WeChat one-on-one chats to adequately address specific or private communication needs, thus enhancing the satisfaction of information communication. In addition, they can compensate for service shortcomings in the community due to insufficient manpower, such as sharing potential emotional care work and providing special services to disadvantaged residents.

### Improving the effectiveness of community risk communication through perceived communication quality

This study incorporates the core concepts of risk perception and perceived communication quality and analyses public satisfaction with community communication during the COVID-19 pandemic. By considering the real situation of community communication in the context of the Shanghai pandemic, this study refines the specific components and verifies their importance. Specifically, this study investigated the coverage of information content provided by community WeChat groups (with dissemination frequency assessing the universality of information dissemination and content adequacy measuring the diversity of information services), as well as perceived response efficiency (evaluating the impact of interactive communication on communication effectiveness from the dimensions of response speed, response frequency, and the frequency of information response after problem resolution). Additionally, while previous research has mostly focused on qualitative analysis of community service content, this study addresses the limitations of research perspectives and methods by using quantitative data to reconfirm the close relationships among risk perception, community service quality, and communication effectiveness.

The significant impact of perceived communication quality on communication satisfaction indicates that using WeChat groups as a digital communication method can effectively improve communication quality. Diverse communication can utilize an open decision-making framework to evaluate options, generate new insights, and enhance the quality of existing knowledge [67]. The interaction among multiple stakeholders helps eliminate perception biases in risk communication, and the transparent presentation of communication content makes residents’ emotional dynamics directly visible.

The most critical factor influencing communication satisfaction is not the response after residents’ problems are solved but rather the response speed (H2-1 is not supported), indicating that WeChat communities need to pay attention to alleviating citizens’ negative emotions in risk communication. Especially for megacities, emphasizing the establishment of trust and the provision of emotional and social support is particularly important. In the context of sudden risks that generate diverse conflicts and needs and the immense population density and base repeatedly test the community’s sustainability and resilience [67], megacities, which are increasingly rising to national and even global centers, are characterized by high risk levels, high media attention, and high public attention [68]. Therefore, once community governance in megacities shows signs of “disintegration”, it is likely to amplify phenomena characterized by risk type transformation or significant public opinion conflicts, leading to a strong social contagion effect.

Therefore, diverse online communication and interactive governance serve as intervention and monitoring mechanisms. The complex demographic context of megacities can not only meet the needs of different people based on different backgrounds but also intervene in negative emotions in a timely manner and foster and solidify residents’ sense of trust and community cohesion. The community built through online WeChat groups promotes public participation through the materiality of digital technology and the immateriality of emotional culture. Core residents can act as a bridge, linking various actors with the emotional power of commonality and public spirit [69]. Even in the face of sudden crisis events or management mistakes, trust serves as a foundation, allowing time for the formulation and implementation of solutions. Additionally, in terms of communication content, it is necessary to strengthen the dissemination of guidance information for postpandemic risk response and enhance popular science components in risk communication. Especially in the face of information overload caused by a large amount of homogeneous information, communities need to change communication strategies and innovative content formats to improve effective communication. In summary, improving the quality and level of community services is crucial for ensuring the effectiveness of risk measures.

### Practical significance and research limitations

In China alone, the number of megacities exceeded 21. The initiative of using community WeChat groups for community governance in Shanghai represents an effective integration of social culture and public governance [70]. It is a successful experiment that is both forward-looking and innovative. Modern global culture exhibits similarities in terms of dissemination and connection, particularly under the development of an “urban society”. Using online familiar social networks for information exchange and emotional maintenance is also one of the current trends in public management [71, 72]. Therefore, considering the integration of social media into community communication is important and has significant practical value.

There are several limitations to this study. First, due to the two-month-long city blockade imposed by the Shanghai municipal government after the 2022 epidemic reoccurred in China, it was difficult to collect data during this period. Although we monitored the results of the questionnaire in real time and proactively contacted and released the questionnaire to community WeChat groups in urban areas with small sample sizes, online snowballing to expand the questionnaire coverage may cause some limitations of the sample. In addition, given that there are individual differences in the level of risk perception among residents, whether the utilization of social media for risk communication in grassroots communities will reduce the risk perception of residents and whether relevant factors such as the amount and frequency of social media information dissemination can be used as variables to moderate the population’s perception of risk to further support public health promotion in the grassroots sector. All of the above factors need to be further studied in the future.

## Conclusion

This study mainly indicates that grassroots community communication based on social media must pay particular attention to rapid responses to messages. At the same time, with the internetization of communication, the inherent pattern of risk communication is broken, and core residents, as new actors in community communication, have the potential to be included in the “semiformal” grassroots governance system. This also opens up new perspectives on the future use of social media for public health monitoring and advocacy, and more attention should be given to the attitudes and needs of residents towards grassroots community work and the use of core residents to cultivate “acquaintance relationships” to maximize the efficiency of communication for community risk prevention and response, thus forming the cornerstone of public health emergencies. Therefore, future studies suggest that mega-city grassroots communities should consider the affordances of social media, recognize the significant correlation between risk communication and grassroots credibility, and formulate more detailed and targeted risk communication strategies to improve the community resilience of grassroots communities facing public health emergencies.

## Data Availability

Data availability statementThe data sets generated during and/or analysed during this study are available from the corresponding author on reasonable request.

## Footnotes

1 Grid-based management: This is an administrative tool that the Chinese government has been increasingly relying on since the COVID-19 outbreak in 2020. The grassroots communities are divided into several grids, and members in a grid use unified urban management and digital platforms to conduct daily affairs and share information about epidemic prevention and control (Wei et al., 2021).

2 Chinese sociologist Fei Xiaotong pointed out that during the traditional rural society period, Chinese people formed the “differential mode of association” through geographically and blood-related interpersonal relationships. In this pattern, morality and ethics served as the bonds maintaining these relationships. People would extend from themselves to others in a “self-centered” manner.

3 According to statistics, the net population inflow rate (the difference between the permanent population and the registered population) in megacities such as Beijing, Shanghai, Guangzhou, and Shenzhen exceeds 35%. Data source: https://mp.weixin.qq.com/s/BHQb--F2iYvQqo9eyzuXMw.

4 According to the “Working Methods of Yangpu District Residents’ Committee” report issued by Shanghai on April 23, 2021, the streets are divided into blocks by building, alley, and house number. Members of the residents’ committee are appointed block leaders and are responsible for contacting and serving the residents.

5 According to the Shanghai Community Council, each community is allocated six volunteer targets during the epidemic prevention and control period, which are unified and registered by the Chinese government. A volunteer service allowance is issued after the target is achieved, and the actual volunteer size is up to 20 people in each community.

6 As a leading megacity, Shanghai has accelerated the adjustment and upgrading of its industrial structure in recent years, attracting a large number of talents. The increase in talent ranks first in China, with nearly 40% holding a bachelor’s degree or higher. Data source: https://www.stats.gov.cn/sj/tjgb/rkpcgb/qgrkpcgb/202302/t20230206_1902006.html.
